# Manifestation of paroxysmal nocturnal hemoglobinuria as repeated acute kidney injury

**Published:** 2015-09-09

**Authors:** Manish R Balwani, Vivek B Kute, Pankaj R Shah, Maulin Shah, Umesh Varyani, Hargovind L. Trivedi

**Affiliations:** ^1^Department of Nephrology and Clinical Transplantation, Institute of Kidney Diseases and Research Center, Dr. HL Trivedi Institute of Transplantation Sciences (IKDRC-ITS), Ahmedabad, India

**Keywords:** Hemolytic anemia, Hemodialysis, Flow cytometry, Paroxysmal nocturnal hemoglobinuria, Acute kidney injury

## Abstract

The triad of hemolytic anemia, pancytopenia, and thrombosis makes paroxysmal nocturnal hemoglobinuria (PNH) a truly unique clinical syndrome. Intravascular hemolysis in PNH can lead to a severe hemolytic episode with massive hemoglobinuria which can cause acute kidney injury (AKI) probably from acute tubular necrosis (ATN). A 15 -year-old girl was admitted with history of fever, diarrhea, vomiting followed by decreased urine output since 3 days. Urinalysis showed nil protein, no red blood cells (RBCs) on microscopy. Plasma hemoglobin level, total leukocyte count, platelet count, and serum creatinine were 6.5 gm/dl, 6440/μl, 205 000/μl, and 3.1 mg/dl, respectively. She received 3 units of packed red blood cells and the patient was discharged with normal renal function test with a diagnosis of acute gastroenteritis with recovered AKI. After 8 months she again had fever, vomiting, nausea with decreased urine output since 3 days. Laboratory investigations showed hemoglobin - 5.5 gm/dl, total leukocyte count - 1550/ μl, platelet count - 165000/μl, and serum creatinine - 4.89 mg/dl. Serum LDH level was 2188 U/l. She was managed conservatively with steroids, antibiotics and she recovered her kidney functions to normal in a week. Presentation of repeated AKI with hemolytic anemia in a short span after fever led us to perform flow cytometric analysis of peripheral blood granulocytes which revealed the presence of PNH clone. PNH may present with renal disease and anemia only even without classical history of hematuria or venous thrombosis. Thus it needs high index of suspicion as early diagnosis and treatment will help in preventing repeated episodes of AKI and thus chronic kidney disease.

**Implication for health policy/practice/research/medical education:**

Paroxysmal nocturnal hemoglobinuria (PNH) may present with renal disease and anemia only even without classical history of hematuria or venous thrombosis. Thus it needs early and high index of suspicion for PNH as early diagnosis and treatment will help in preventing repeated episodes of acute kidney injury (AKI) and thus chronic kidney disease (CKD).

## Introduction

The triad of hemolytic anemia, thrombosis and pancytopenia makes paroxysmal nocturnal hemoglobinuria (PNH) a truly unique clinical syndrome. Intravascular hemolysis in PNH can lead to two forms of renal disease: 1) a severe hemolytic episode of any cause with massive hemoglobinuria can cause acute kidney injury (AKI) probably from acute tubular necrosis (ATN), and 2) chronic hemolysis results in iron deposition in the kidneys in almost all patients. Timely diagnosis and management of PNH will prevent progression of kidney disease and complications of PNH.

## Case presentation

A 15-year-old girl was admitted to our center with history of fever, diarrhea, nausea and vomiting followed by decreased urine output since three days. She gave history of menorrhagia since last 2 months. There was no history of abdominal pain, skin rashes, reddish urine, joint pains, edema feet or intake of any medications. On examination, she had pallor, no edema. Systemic examination was normal. Past history was unremarkable. Urinalysis showed nil protein, no red blood cells (RBCs) on microscopy, hemoglobin - 6.5 gm/dl, total leukocyte count - 6440/µl, platelet count - 205000/µl. Urine for hemoglobin and myoglobin were negative. Serum creatinine was 3.1 mg/dl. Serum total bilirubin was 1.7 mg/dl (indirect level; 0.9 mg/dl). Serum AST, ALT, alkaline phosphatase and serum albumin were normal. Serum LDH level was 1500 U/l. Chest x-ray and ultrasonography of abdomen were normal. Serum ANA, anti-ds DNA and ASO titer were negative and urine culture sterile. Serum complement levels were normal. Peripheral smear showed mild to moderate anisopoikilocytosis, and polychromasia. The reticulocyte count was 1.6%. Osmotic fragility, hemoglobin electrophoresis and G6PD levels were normal. Coombs test was negative. Patient was managed symptomatically and the renal function improved after admission. She received three units of packed red blood cells and the patient was discharged with normal renal function test with a provisional diagnosis of acute gastroenteritis with recovered AKI. She again came to our hospital after 8 months with history of fever, vomiting, nausea with decreased urine output since three days. She had menorrhagia again. On examination, she had pallor, no edema. Blood pressure was 126/80 mm Hg and systemic examination was unremarkable. Laboratory parameters: serum hemoglobin - 5.5gm/dl, total leukocyte count - 1550/ µl, platelet count - 165000/µl, and serum creatinine - 4.89 mg/dl. Serum total bilirubin was 1.2 mg/dl (indirect was 1.0 mg/dl). Serum AST, ALT, alkaline phosphatase and serum albumin were normal. Serum LDH level was 2188 U/l. Peripheral smear showed mild to moderate anisopoikilocytosis, schistocytes and polychromasia. She required two sessions of hemodialysis in which 3 packed red cell transfusion was given. She was further managed conservatively with steroids, antibiotics and she recovered her kidney functions to normal in a week. We did not performed a kidney biopsy as she recovered both times in short duration. Presentation of repeated AKI with hemolytic anemia in such a short span after fever led us to perform flow cytometric analysis of peripheral blood granulocytes which revealed the presence of a PNH clone. The diagnosis of PNH as a cause of hemolytic anemia was confirmed.

## Discussion

The incidence of AKI related to hemolysis is not well described, may be up to 50% with massive hemolysis (1). PNH is one of a cause of intravascular hemolysis. PNH is a disorder characterized by a defect in the glycosylphosphatidylinositol (GPI) anchor due to an abnormality in the *Pig-a* gene. This leads to partial or complete absence of certain GPI-linked proteins, particularly CD59 and CD55 (decay accelerating factor) (2). The triad of hemolytic anemia, thrombosis and pancytopenia makes PNH a truly unique clinical syndrome. Intravascular hemolysis in PNH can lead to two forms of renal disease: a severe hemolytic episode of any cause (often in association with gastroenteritis) with massive hemoglobinuria can cause AKI probably from ATN (3), and chronic hemolysis results in iron deposition in the kidneys in almost all patients. Chronic renal failure due to hemosiderosis and interstitial scarring may occur in patients with long-standing PNH (4). Hemoglobinuria is intermittent in most patients and never occurs in some as was seen in our patient, but hemosiderinuria is commonly seen. Electron microscopy of renal parenchyma confirms the loss of proximal tubular brush borders and demonstrates decreased infoldings at the basolateral membrane of proximal tubular epithelial cells (5). Flow cytometric analysis of peripheral blood cells (granulocytes or RBCs) using antibodies directed against GPI anchored proteins (GPI-AP) is the most sensitive and informative assay for diagnosis of PNH (6). Our patient presented twice with markedly deranged renal functions with very high creatinine level and features suggestive of intravascular hemolytic anemia. There are case reports that show occurrence of AKI and chronic kidney disease (CKD) due to PNH. In all these cases a diagnosis of PNH was made after detection of renal dysfunction as was in our case (5). General goals for preventive therapy in heme pigment induced acute renal failure are correcting volume depletion and preventing intratubular cast formation. Early recognition of AKI and its immediate management with fluid resuscitation is important as persistent kidney injury predisposes to long-term consequences (7). The only potentially curative treatment for PNH is allogeneic hematopoietic cell transplantation. Treatment comprises transfusion therapy in the acute phase and correction of concomitant iron deficiency. Corticosteroids may have a role in attenuating acute hemolytic exacerbations and it was given for short period in our patient at the time of hemolytic episode. For selected transfusion-dependent patients or patients with disabling symptoms (e.g. fatigue, thromboses, frequent paroxysms of pain, end-organ damage), treatment with eculizumab is suggested (8). Our patient was discharged on iron supplement and folic acid.

## Conclusion

PNH presenting as recurrent acute renal failure is extremely rare. This case has been reported to highlight a rare, but potentially reversible cause of recurrent acute renal failure. Early diagnosis and treatment are crucial to prevent disease progression and irreversible CKD.

## Authors’ contribution

All authors wrote the paper equally.

## Conflicts of interest

The authors declared no competing interests.

## Ethical considerations

Ethical issues (including plagiarism, data fabrication, double publication) have been completely observed by the authors.

## Funding/Support

None.
